# CryoEM analysis of small plant biocatalysts at sub-2 Å resolution

**DOI:** 10.1107/S205979832101216X

**Published:** 2022-01-01

**Authors:** Nicole Dimos, Carl P. O. Helmer, Andrea M. Chánique, Markus C. Wahl, Robert Kourist, Tarek Hilal, Bernhard Loll

**Affiliations:** a Institute of Chemistry and Biochemistry, Department of Biology, Chemistry, Pharmacy, Laboratory of Structural Biochemistry, Free University of Berlin, Takustrasse 6, 14195 Berlin, Germany; b Institute of Molecular Biotechnology, Graz University of Technology, Petersgasse 14, 8010 Graz, Austria; c Department of Chemical and Bioprocesses Engineering, School of Engineering, Pontificia Universidad Católica de Chile, Vicuña Mackenna 4860, 7810000 Santiago, Chile; d Macromolecular Crystallography, Helmholtz-Zentrum Berlin für Materialien und Energie, Albert-Einstein-Strasse 15, 12489 Berlin, Germany; e Institute of Chemistry and Biochemistry, Research Center of Electron Microscopy and Core Facility Bio­SupraMol, Free University of Berlin, Fabeckstrasse 36A, 14195 Berlin, Germany; f moloX GmbH, Takustrasse 6, 14195 Berlin, Germany

**Keywords:** cryo-electron microscopy, camphor, terpenes, borneol dehydrogenases, high resolution, green chemistry, plant biocatalysts

## Abstract

Enzyme catalysis has emerged as a key technology for developing efficient, sustainable processes in the chemical, biotechnological and pharmaceutical industries. Plants provide large and diverse pools of biosynthetic enzymes that facilitate complex reactions, such as the formation of intricate terpene carbon skeletons, with exquisite specificity. High-resolution structural analysis of these enzymes is crucial in order to understand their mechanisms and modulate their properties by targeted engineering. Although cryo-electron microscopy (cryoEM) has revolutionized structural biology, its applicability to high-resolution structural analysis of comparatively small enzymes has so far been largely unexplored. Here, it is shown that cryoEM can reveal the structures of plant borneol dehydrogenases of ∼120 kDa at or below 2 Å resolution, paving the way for the rapid development of new biocatalysts that can provide access to bioactive terpenes and terpenoids.

## Abstract 2

Although cryo-electron microscopy has revolutionized structural biology, its applicability to high-resolution structural analysis of comparatively small enzymes so far remains largely unexplored. Here, two cryo-EM structures of plant borneol dehydrogenases of ∼120 kDa at or below 2 Å resolution are reported.

## 1. Introduction

Despite the stunning recent success of single-particle cryoEM in the structural analysis of many large molecular machines, comparatively small proteins remain a major challenge for this technique (Kühlbrandt, 2014 ▸; Lyumkis, 2019 ▸; Vinothkumar & Henderson, 2016 ▸). To date, the highest resolution achieved by cryoEM is 1.14 Å for the highly symmetric 480 kDa protein apoferritin (Yip *et al.*, 2020 ▸). Presently, the cryoEM structures of only four macromolecular complexes smaller than 120 kDa have been reported at a resolution better than 3.0 Å (Supplementary Table S1). Due to limited structural data and frequently insufficient understanding of the molecular basis of enzyme catalysis, protein engineering still mainly relies on combinatorial approaches for enzyme engineering such as random or saturation mutagenesis. An expansion of the scope of single-particle analysis towards the rapid elucidation of the structures of smaller proteins has tremendous potential to increase the rational element of protein engineering.

Enzyme catalysis offers an efficient and sustainable alternative to traditional chemical synthesis, as biocatalysts harbor excellent selectivity and work under mild reaction conditions. Today, enzymes are widely used in the chemical and pharmaceutical industries, and the application of biocatalysts to the manufacture of chemicals from renewable resources is a rapidly growing field. However, due to limited structural data and, as a consequence, insufficient understanding of the molecular basis of enzyme catalysis, rational improvement of biotechnologically relevant enzymes has been severely hampered. Protein engineering relies mainly on directed evolution or semi-rational approaches. While often successful, these methods require the implementation of high-throughput screenings and a substantial effort in terms of laboratory work, leading to long time-to-market horizons. This situation could be alleviated by expanding the scope of cryoEM towards the elucidation of high-resolution structures of smaller proteins.

A particularly interesting application of enzyme catalysis is the synthesis and modification of bioactive terpenes and terpenoids. With more than 50 000 different structures, terpenes are a structurally and functionally extraordinarily diverse group of natural products (Oldfield & Lin, 2012 ▸). The outstanding selectivity of the enzymes involved in the formation of terpene carbon skeletons (Christianson, 2017 ▸), their primary functionalization (Bohlmann & Keeling, 2008 ▸) and their further derivatization (Rinkel *et al.*, 2019 ▸) could enable the formation of a myriad of new terpene derivatives with diverse, interesting properties for the food and pharmaceutical industries via environmentally friendly catalytic processes (Newman & Cragg, 2016 ▸; Oldfield & Lin, 2012 ▸). To this end, a detailed understanding of the molecular basis of the reaction mechanisms and selectivity of the enzymes is required.

Bornane-type monoterpenoids, such as borneol, isoborneol and camphor, are found in essential oils from plants and are used in traditional medicine and cosmetics (Cheng *et al.*, 2013 ▸). Racemic borneol, isoborneol and camphor are currently produced from α-pinene, a side product of cellulose production. Essential oils from plants are often enriched in one of the enantiomers of these compounds, indicating the potential presence of highly stereoselective borneol dehydrogenases (BDHs). An enzymatic route towards pure enantiomers using enantioselective dehydrogenases would be highly desirable to avoid the labor-intensive and expensive extraction of pure enantiomers from plants.

**Figure 1:**
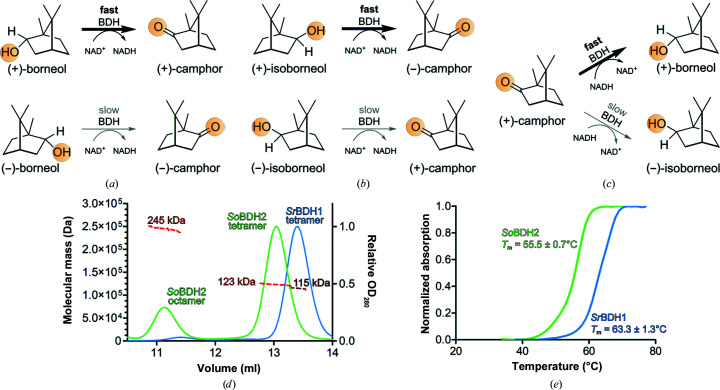
Reaction schemes and enzyme characterization of *So*BDH2 and *Sr*BDH1. The BDHs discussed here preferentially convert the (+)-enantiomers of borneol and isoborneol. *Sr*BDH1 is highly selective for both alcohols and catalyzes the reduction of (+)-camphor. *So*BDH2 is highly selective for borneol. *Ps*BDH only shows a slight selectivity for both borneol and isoborneol and is capable of catalyzing the reduction of camphor (Khine *et al.*, 2020 ▸). (*a*, *b*) Reaction schemes of *Sr*BDH1 and *So*BDH2 in the enantiospecific oxidation of *rac*-borneol (*a*) and *rac*-isoborneol (*b*). (*c*) Reduction of (+)-­camphor. (*d*) SEC-MALS analysis of *Sr*BDH1 (blue) and *So*BDH2 (green). For *Sr*BDH1 a single peak is observed consistent with a tetramer (theoretical molecular mass 120 kDa). The first peak in the chromatogram of *So*BDH2 corresponds to an octamer (theoretical molecular mass 258 kDa) and the second peak corresponds to a tetramer (theoretical molecular mass 129 kDa). The brown (*Sr*BDH1) and red (*So*BDH2) curves are refractive-index signals. (*e*) Differential scanning fluorometry reveals a significantly lower *T*_m_ for SoBDH2 (55.5 ± 0.7°C) compared with *Sr*BDH1 (63.3 ± 1.3°C).

BDHs belong to the family of short-chain dehydrogenase–reductases (SDRs; Chánique *et al.*, 2021 ▸). The members of this enzyme class have a TG*XXX*(AG)*X*G NAD^+^-binding motif and a Y*XXX*K active-site motif (Ladenstein *et al.*, 2008 ▸; Kallberg *et al.*, 2002 ▸) and form dimers or tetramers. Some BDHs have a high twofold stereoselectivity in the conversion of chiral monoterpenoids (Chánique *et al.*, 2021 ▸; Croteau *et al.*, 1978 ▸; Drienovská *et al.*, 2020 ▸) by preferring one of the two substrate enantiomers and forming a stereocenter by asymmetric reduction of the diastereotopic keto group. The selective oxidation of (+)-borneol to (+)-camphor by a partially purified BDH from *Salvia officinalis* L. was first described by Croteau *et al.* (1978 ▸). Recently, the isolation and purification of two BDHs confirmed the high stereoselectivity of these enzymes (Figs. 1 ▸ *a*, 1 ▸ *b* and 1 ▸ *c*; Drienovská *et al.*, 2020 ▸; Chánique *et al.*, 2021 ▸).

The understanding of terpene formation on a structural and mechanistic basis is important for the engineering of biosynthetic pathways for the formation of new terpenoids (Kemper *et al.*, 2017 ▸). Unfortunately, the difficulty in producing enzymes from higher organisms in bacteria, and often their limited stability, make structure determination by classical crystallization very challenging. In order to obtain a structure under these circumstances, approaches such as truncation and homology modeling have been utilized, both of which have limited informational value for mechanistic studies and enzyme engineering. In the particular case of BDHs, only two crystal structures have been reported to date: those of the nonselective bacterial BDH from *Pseudomonas* sp. TCU-HL1 (*Ps*BDH; PDB entry 6m5n; Khine *et al.*, 2020 ▸) and the enantioselective BDH from *Salvia rosmarinus* (*Sr*BDH1; Chánique *et al.*, 2021 ▸). Although structural analysis of *Sr*BDH1 allowed us to identify a hydrophobic pocket that discriminates the monoterpenol isoborneol, structures of additional BDHs, for example from *S. officinalis* (*So*BDH2), are required to rationalize the selectivity of the enzymes towards (+)-borneol. Here, we report the determination of the structures of two stereoselective dehydrogenases, *Sr*BDH1 and *So*BDH2, by single-particle cryoEM.

## 2. Experimental procedures

### 2.1. Cloning

The synthetic genes for the borneol-type dehydrogenases *So*BDH2 from *S. officinalis* (GenBank ID MT525099) and *Sr*BDH1 from *S. rosmarinus* (GenBank ID MT857224) were ordered from GenScript (USA), codon-optimized for *Escherichia coli* expression and cloned into the vector pET-15b in frame with an N-terminal His_6_ tag (Chánique *et al.*, 2021 ▸; Drienovská *et al.*, 2020 ▸).

### 2.2. Expression and purification of *So*BDH2 

*E. coli* BL21-RIL cells (Stratagene) were transformed with a pET-15a vector containing *So*BDH2 fused to an N-terminal hexahistidine tag. Protein induction was carried out in auto-induction medium at 37°C for 7 h with subsequent cooling to 16°C (Studier, 2018 ▸). The cells were grown overnight and harvested by centrifugation (10 min at 7000 rev min^−1^ at 4°C). The pellets were resuspended in 20 m*M* Tris–HCl pH 8.0, 500 m*M* NaCl (buffer *A*). The cells were lysed by homogenization at 4°C for 7 min after the addition of 0.5 mg l^−1^ DNase and the lysate was cleared by centrifugation (30 min at 21 500 rev min^−1^ at 4°C). All subsequent purification steps were performed at 4°C. A Ni^2+^–NTA column (1 ml column volume, Macherey Nagel) was equilibrated with buffer *A*, and *So*BDH2 was loaded onto the column and washed with 15 column volumes of buffer *A*. *So*BDH2 was eluted with buffer *A* supplemented with 300 m*M* imidazole. The protein was incubated with a threefold molar excess of NAD^+^ (0.5 *M* in double-distilled H_2_O) for 10 min on ice prior to size-exclusion chromatography (SEC). SEC was performed using a HiLoad Superdex S200 16/60 column (GE Healthcare) equilibrated with 20 m*M* Tris–HCl pH 8.0, 125 m*M* NaCl. Pooled protein fractions were concentrated with Amicon Ultra-15 (Merck KGaA) to 11.2 mg ml^−1^ as measured by the absorbance at 280 nm. *Sr*BDH1 was purified using a practically identical protocol (Chánique *et al.*, 2021 ▸).

### 2.3. Size-exclusion chromatography–multi-angle light scattering (SEC-MALS)

SEC-MALS experiments were performed at 18°C. *So*BDH2 was loaded onto a Superdex 200 Increase 10/300 column (GE Healthcare) coupled to a miniDAWN TREOS three-angle light-scattering detector (Wyatt Technology) in combination with a RefractoMax520 refractive-index detector. For calculation of the molecular mass, protein concentrations were determined from the differential refractive index with a specific refractive-index increment (d*n*/d*c*) of 0.185 ml^−1^. Data were analyzed using the *ASTRA* 6.1.4.25 software (Wyatt Technology).

### 2.4. Differential scanning fluorometry (DSF)

The melting temperatures of the proteins were measured using an Mx3005P qPCR system (Agilent) in 96-well plate format under the buffer condition 20 m*M* Tris–HCl pH 8.0, 125 m*M* NaCl as used for crystallization or cryoEM experiments. Each well contained 10 µl buffer and 10 µl protein (0.15 µg µl^−1^) with a final concentration of 10× SYPRO Orange dye (Invitrogen). The program consisted of three steps: step 1 was a pre-incubation for 1 min at 20°C and steps 2 and 3 were cycles comprising a temperature increase of 1°C within 20 s. The temperature gradient proceeded from 25 to 95°C at 1°C per minute. Samples were measured in triplicate. The data were acquired using the *MxPro QPCR* software (Agilent) and analyzed using the *DSF Analysis* version 3.0.1 tool (ftp://ftp.sgc.ox.ac.uk/pub/biophysics) and *GraphPad Prism* 5.0.0.228 (Graph Pad Software). A *t*-test was performed with *GraphPad Prism* to validate the significance of the results.

### 2.5. Cryo-electron microscopy

Samples were diluted to 1 mg ml^−1^ and a total of 3.8 µl was applied onto glow-discharged 300 mesh holey gold UltrAuFoil R1.2/1.3 grids (Quantifoil Micro Tools GmbH). Vitrification was conducted using a Vitrobot Mark IV (Thermo Fisher Scientific, Eindhoven, The Netherlands) set to 10°C and 100% humidity by plunging into liquid ethane after 4 s of blotting.

Data for *So*BDH2 were collected on an FEI Titan Krios G3i transmission electron microscope (Thermo Fisher Scientific, Eindhoven, The Netherlands) operated at 300 kV equipped with a Falcon 3EC at a nominal magnification of 96 000×, corresponding to a calibrated pixel size of 0.832 Å. Objective astigmatism and coma were corrected with *AutoCTF* (Thermo Fisher Scientific, Eindhoven, The Netherlands) under the final imaging conditions. To maximize beam coherence, a 50 µm C2 aperture was chosen. Direct alignments were executed thoroughly and beam parallelism and condenser astigmatism were optimized using the ronchigram on a Volta phase plate (VPP), which was retracted during data acquisition. During imaging an electron flux of 0.7 e^−^ per pixel per second on the detector was selected, corresponding to an exposure rate of 1 e^−^ Å^−2^ s^−1^ on the sample. Images were taken at a nominal defocus of between −0.6 and −1.6 µm, accumulating a total electron exposure of 40 e^−^ Å^−2^ during a 40 s exposure, fractionated into 33 images. For automated data acquisition, *EPU* 2.8.1 (Thermo Fisher Scientific, Eindhoven, The Netherlands) was utilized with aberration-free image shift (AFIS) enabled, allowing 6 µm image-beam-shift acquisition. The implemented ice filter was adjusted to exclusively image regions with the thinnest ice.

Data for *Sr*BDH1 were acquired on the same instrument with minor exceptions. The nominal magnification was increased to 120 000×, yielding a pixel size of 0.657 Å. The electron flux was adjusted to 0.6 e^−^ per pixel per second on the detector, resulting in a dose rate of 1.3 e^−^ Å^−2^ s^−1^ on the sample. During an exposure time of 31 s, a total dose of 40 e^−^ Å^−2^ was applied to the sample.

### 2.6. CryoEM image processing

Raw movies of the *So*BDH2 data set were aligned and dose-weighted with patch-motion correction implemented in *cryoSPARC* version 2.9 (Punjani *et al.*, 2017 ▸). Initial CTF estimation was achieved using *Patch CTF*. For initial particle picking, the *Blob Picker* was used with a particle diameter of 120–160 Å. Shiny class averages generated by reference-free 2D classification were selected as templates for template-based particle picking using a 120 Å circular mask. A total of 1 551 724 particle images were extracted with a box size of 224 pixels Fourier-cropped to 56 pixels (3.328 Å per pixel) for initial analysis and subjected to 40 iterations of 2D classification. Shiny classes were selected for *ab initio* reconstruction imposing *D*2 symmetry. Heterogeneous refinement with three classes did not guide further classification; therefore, particle images were re-extracted Fourier-cropped to a box size of 112 pixels (1.664 Å per pixel). The best resolved structure after heterogeneous refinement was re-extracted with a box size of 256 pixels (0.832 Å per pixel). Non-uniform (NU) refinement into a single class of 290 356 particles yielded a reconstruction with 2.32 Å resolution. Global and local CTF correction did not improve the resolution; however, the reconstruction visually appeared to be better defined. In order to better account for anisotropic motion of the particles, local motion correction was applied followed by global CTF refinement, yielding a reconstruction after NU refinement at 2.2 Å resolution. Micrographs with estimated resolutions of worse than 3.5 Å were discarded, leaving 254 403 particle images for another cycle of local motion correction followed by global CTF refinement and NU refinement. To account for the point spread of the signal in the particle images, a box size of 384 pixels (320 Å) was used for re-extraction, giving a resolution after NU refinement of 2.1 Å. Another heterogeneous refinement run was conducted to isolate the final population of 173 781 particle images, which was reconstructed after local motion correction by NU refinement to 2.0 Å resolution. In the later NU refinement runs, references were initially filtered to 20 Å to retain more structural information in the reference projections, which helped to stabilize refinement. We suspect that the similar appearance of BDH from perpendicular projections of the top view exacerbates the alignment which results in misaligned particles, thus limiting the resolution.

*Sr*BDH1 was refined similarly, with the exception that choosing the same final box size of 384 pixels resulted in smaller absolute dimensions of the box. From a total of 1587 micrographs 1 635 690 particle images were extracted, resulting in 410 573 selected particle images after reference-free 2D classification. After iterative homogeneous and heterogeneous refinement cycles, a final subset of 210 505 particle images were selected, yielding a reconstruction with 1.88 Å resolution after NU refinement.

### 2.7. Model building and refinement

An initial model of *So*BDH2 was obtained by automatic model building with *ARP*/*wARP* *ARPEM* (version 8.0; Chojnowski *et al.*, 2019 ▸) using the protein sequence as input and a sharpened Coulomb potential map. Sharpening was achieved by density modification with *phenix.resolve_cryo_em* with default settings using unfiltered, unmasked half-maps and the nominal resolution determined by gold-standard FSC. Sharpening of the *Sr*BDH1 reconstruction was conducted with *phenix.auto_sharpen* using default settings starting with unfiltered, unmasked half-maps and the gold-standard resolution as the target resolution. Automatic model building comprised iterative refinement in *REFMAC*5 (version 5.8.0258; Murshudov *et al.*, 2011 ▸). For comparison, the *phenix.map_to_model* procedure (Terwilliger *et al.*, 2018 ▸) as well as *Buccaneer* (Hoh *et al.*, 2020 ▸) as part of the *CCPEM* suite (Burnley *et al.*, 2017 ▸) were used for automated model building. The automated model-building programs were run with the standard settings, since they gave the best results. The obtained model was manually adjusted to the cryoEM density, supported by real-space refinement in *Coot* (version 0.8.9.1; Casañal *et al.*, 2020 ▸). The model was refined against the cryoEM map using the real-space refinement protocol in *Phenix* (version 1.19.1; Liebschner *et al.*, 2019 ▸; Afonine *et al.*, 2018 ▸). Water molecules were added in *Coot* and manually inspected, followed by an additional round of real-space refinement in *Phenix*. In the final stages of refinement, we fully released the restraints for secondary-structure elements, Ramachandran, noncrystallographic symmetry (NCS) and no corrections of energetically disfavored rotamer conformations. In final rounds of refinement, grouped atomic displacement factors were refined. The structures were evaluated with *EMRinger* (Barad *et al.*, 2015 ▸) and *MolProbity* (Williams *et al.*, 2018 ▸). Structure figures were prepared using *PyMOL* (version 1.8; Schrödinger) and *UCSF Chimera* (Pettersen *et al.*, 2004 ▸). Secondary-structure elements were assigned with *DSSP* (Kabsch & Sander, 1983 ▸), and *ALSCRIPT* (Barton, 1993 ▸) was used for secondary-structure-based sequence alignments. The atomic models have been deposited in the Protein Data Bank (PDB) with the following accession codes: 7o6p for the 2.04 Å resolution structure of *So*BDH2 and 7o6q for the 1.88 Å resolution structure of *Sr*BDH1. The cryoEM maps have been deposited in the Electron Microscopy Data Bank as follows: *So*BDH2, EMD-12739; *Sr*BDH1, EMD-12740.

## 3. Results and discussion

### 3.1. High-resolution cryoEM structure of *So*BDH2

*Sr*BDH1 and *So*BDH2 exhibit 44% sequence identity and 60% sequence similarity. We produced *So*BDH2 with an N-terminal His_6_ tag (theoretical molecular mass 32.2 kDa) in *E. coli* and prepared the protein at high purity. Size-exclusion chromatography coupled to multi-angle light scattering (SEC-MALS) revealed two distinct species (Fig. 1 ▸ *d*) corresponding to an octameric and a tetrameric assembly.

Encouraged by the possible occurrence of an octameric assembly, we considered cryoEM as powerful method to dissect structural heterogeneity, and prepared cryoEM grids. Imaging was conducted on a Titan Krios 300 kV TEM equipped with a Falcon 3EC detector operated in counting mode. We aligned the instrument thoroughly and aimed to maximize the beam coherence by choosing a 50 µm C2 aperture. To optimize the C2 intensity and stigmation, we used the ronchigram method on a Volta phase plate (VPP; Rodenburg & Macak, 2002 ▸). The VPP was only used for alignment and was retracted during data acquisition. A total of 1439 micrographs was acquired and subjected to motion correction and CTF estimation. From the 1 551 724 particle images that were initially picked, 173 781 particle images were selected by iterative 2D and 3D classification cycles for homogeneous 3D refinement (Supplementary Figs. S1 and S2). Although we had observed a fraction of octamers in solution (Fig. 1 ▸ *d*), 3D refinement only yielded a tetrameric structure (Supplementary Fig. S2); we also failed to detect octamers in negative-stain EM.

**Figure 2:**
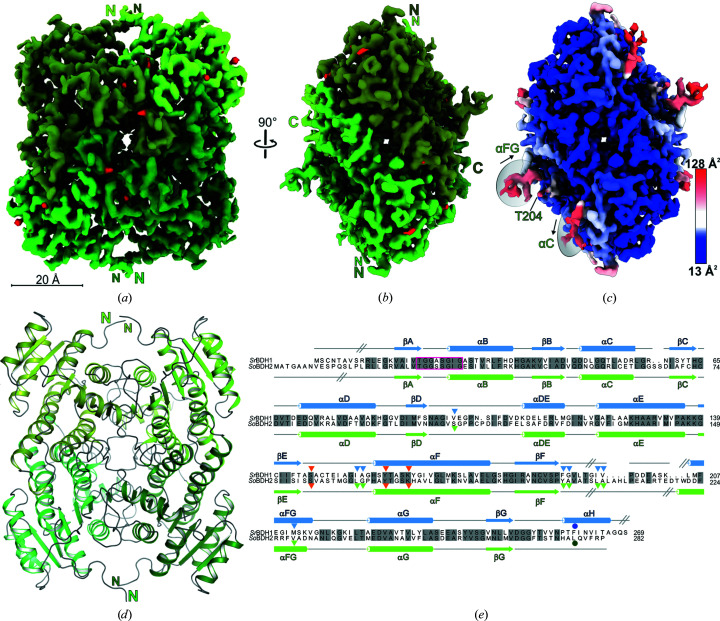
CryoEM structure of *So*BDH2. (*a*) Tetrameric assembly of *So*BDH2. Density at a contour level of 1.1 is shown for each of the four protomers in different shades of green after sharpening with *Phenix*. The locations of very well defined water molecules are shown in red. (*b*) The same color-coding as in (*a*), but rotated by 90°. (*c*) Grouped *B* factor mapped onto the density. The color gradient is from blue to red corresponding to increasing *B* factors. Regions with high *B* factors are highlighted with gray ellipses and are labeled according to the assigned secondary structure. (*d*) *So*BDH2 structure in cartoon representation. The same view and color-coding as in (*a*) is used. (*e*) Structure-based sequence alignment of *Sr*BDH1 (GenBank ID MT857224) and *So*BDH2 (GenBank ID MT525099) as obtained by cryoEM. Secondary-structure elements are drawn above the alignment for *Sr*BDH1 and below the alignment for *So*BDH2, with α-helices depicted as cylinders and β-strands as arrows. Gray inclined lines indicate sections of the structures which could not be modeled since they were not resolved in the reconstruction. Orange triangles indicate the catalytic motif. Amino acids lining the putative active site of *Sr*BDH1, based on its crystal structure (PDB entry 6zyz) with bound NAD^+^, are indicated by blue triangles and by a dark blue circle if derived from the C-terminal portion of another *Sr*BDH1 monomer within the tetramer. The dark green circle marks a residue derived from another protomer of *So*BDH2 that completes the active site. Gray-shaded amino acids are identical. The TG*XXX*(AG)*X*G NAD^+^-binding motif, between βA and αB, is indicated with a magenta rectangle.

After the application of global and local CTF refinement, particle-based local motion correction and NU refinement within the *cryoSPARC* framework (Punjani *et al.*, 2017 ▸), a final gold-standard resolution of 2.04 Å was obtained. The obtained cryoEM density reflects the nominal resolution, as individual side chains could be unambiguously identified and built. Given the high resolution of our cryoEM map (Figs. 2 ▸ *a* and 2 ▸ *b* and Table 1 ▸), we tested how the automated model-building programs *ARP*/*wARP* (Chojnowski *et al.*, 2019 ▸), *phenix.map_to_model* (Terwilliger *et al.*, 2018 ▸) and *Buccaneer* (Hoh *et al.*, 2020 ▸) would perform. The programs were run with the recommended standard settings and the results are summarized in Supplementary Table S2. All programs managed to fit large portions of the protein sequence to the density (82–92%), with *ARP*/*wARP* outperforming the other two programs. We manually completed the initial *ARP*/*wARP* model. Spherical density regions clearly indicated water molecules, and well defined water molecules were automatically placed with *Coot* (Casañal *et al.*, 2020 ▸). The quality of the density allowed the modeling of 50 double conformations of amino-acid side chains and the localization of 268 water molecules. The final model exhibits an excellent fit to the density, with mask/volume correlation coefficients of 0.86/0.83 (Table 1 ▸).

As previously observed in the crystal structures of *Sr*BDH1 and *Ps*BDH, the *So*BDH2 homotetramer exhibits *D*2 symmetry (Fig. 2 ▸ and Supplementary Fig. S1). The protomers adopt a Rossmann-like fold (Rossman *et al.*, 1975 ▸) as required for binding of the NAD^+^ cofactor (Supplementary Fig. S3). The 12 N-terminal residues and the preceding His_6_ tag lack density (Fig. 2 ▸ *e*). Very weak and fragmented density is observed for *So*BDH2 residues Gln52–Gly65 that fold into α-helix αC (Fig. 2 ▸ *e*), reflected by elevated *B* factors (Fig. 2 ▸ *c*). Moreover, the region from Ser205 to Glu218 is not resolved in the density and has not been modeled (Fig. 2 ▸ *e*), which is in agreement with the observation that we could not observe any density for the NAD^+^ cofactors in their binding pockets. The latter observation is in agreement with the apo-state crystal structure of *Sr*BDH1. However, the crystal structure of apo *Sr*BDH1 could only be obtained after co-crystallization with the substrate (+)-borneol, which led to the reduction of NAD^+^ and the release of product and cofactor. Loss of the cofactor could not be prevented by adding a threefold molar excess of NAD^+^ to *So*BDH2 before size-exclusion chromatography. While the loss of NAD^+^ may have occurred during vitrification of the cryoEM sample, in the NAD^+^-bound crystal structures of *Sr*BDH1 the cofactor-binding site is stabilized by crystal contacts, suggesting that under the crystallization conditions the NAD^+^-binding site is artificially stabilized to prevent release of the cofactor.

### 3.2. Active site of *So*BDH2

**Figure 3:**
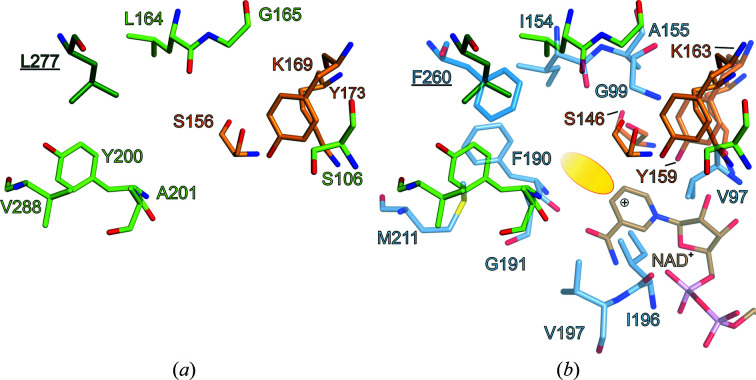
Active-site architecture of *So*BDH2. Residues of the catalytic motif are colored orange and residues of *So*BDH2 lining the active site are colored light green. The substrate-binding pocket is completed by Leu277 (underlined) from the other, neighboring protomer. (*a*) Substrate-binding pocket of *So*BDH2. Numbering refers to residues of *So*BDH2. (*b*) Superposition of the cryoEM structure of *So*BDH2 and the crystal structure of *Sr*BDH1–NAD^+^ (PDB entry 6zyz; Chánique *et al.*, 2021 ▸). Residues of *Sr*BDH1 are drawn in light blue or marine for Phe260 (underlined) from the other, neighboring protomer. The numbering of amino acids refers to *Sr*BDH1. Residues in the equivalent positions to Ile196 and Val197 in *Sr*BDH1–NAD^+^ are not resolved in the density of *So*BDH2 due to the absence of NAD^+^. The corresponding residues to the latter two residues in *So*BDH2 are Leu206 and Ala207, respectively. The yellow ellipse indicates the potential substrate-binding site.

Despite the absence of NAD^+^, the spatial arrangement of the catalytic Ser156, Lys169, Tyr173 motif (Fig. 2 ▸ *e*) is maintained in *So*BDH2 compared with *Sr*BDH1–NAD^+^ (PDB entry 6zyz; Chánique *et al.*, 2021 ▸; Fig. 3 ▸). The lysine residue, in concert with the positively charged nicotinamide, lowers the p*K*_a_ value of the tyrosine, which acts as the catalytic acid/base. The serine residue is involved in stabilization and polarization of the carbonyl function of the substrate (Kavanagh *et al.*, 2008 ▸). As in SrBDH1, the substrate-binding niche is very hydrophobic, but is decorated by different amino-acid residues. Moreover, in both enzymes the C-terminus of another protomer completes the active-site pocket (Fig. 3 ▸). Notably, the C-terminus of *So*BDH2 adopts a coiled-coil structure, in contrast to the C-terminal α-helix αH in *Sr*BDH1 (Supplementary Fig. S7*a* ), but both Phe260 of *Sr*BDH1 and Leu277 of *So*BDH2 reside in the same position (Fig. 3 ▸ *b*).

Due to fold differences, the active-site architectures of plant BDHs and *Ps*BDH differ drastically (Supplementary Fig. S7*c* ). In both *So*BDH2 and *Sr*BDH1 the single αFG helix flanks the substrate-binding site, while the equivalent region in *Ps*BDH is divided into two discrete helices (Supplementary Fig. S7*c* ): αFG1 and αFG2. Furthermore, the C-terminus of *Ps*BDH does not contribute to the substrate-binding site. The differences could be related to the natural functions of the enzymes. The bacterial enzyme, in contrast, participates in the degradation of monoterpenols. Development of stereoselectivity in a catabolic dehydrogenase would restrict the substrate scope as some potential substrates can no longer be converted. While catabolic enzymes generally have a broader substrate acceptance than their anabolic counterparts, in this particular case the development of enantiospecificity would preclude the oxidation of both enantiomers of borneol and isoborneol. As both the (+)- and (−)-enantiomers of these two terpenoids are constituents of the essential oils of many plants, it can be argued that the development of stereoselectivity does not provide an evolutionary advantage.

### 3.3. CryoEM structure of *Sr*BDH1

To explore the general applicability of cryoEM to the high-resolution structural analysis of small plant enzymes, we also subjected *Sr*BDH1 to cryoEM-based structure analysis. The *Sr*BDH1 preparation yielded a single peak in a SEC-MALS analysis, consistent with a tetramer in solution, in agreement with its crystal structure (Chánique *et al.*, 2021 ▸). As *Sr*BDH1 readily crystallized under various conditions, unlike *So*BDH2, we compared the thermal stabilities of the two proteins by differential scanning fluorometry. Interestingly, the readily crystallizable *Sr*BDH1 is stabilized by approximately 8°C compared with *So*BDH2 (Fig. 1 ▸ *e*).

**Figure 4:**
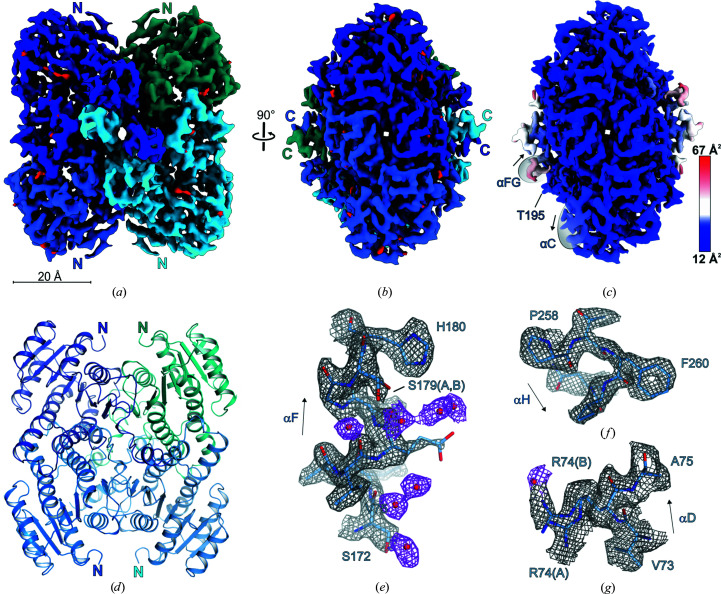
CryoEM structure of *Sr*BDH1. (*a*) Tetrameric assembly of *Sr*BDH1. Density-modified cryoEM reconstructions at a contour level of 9.5 are shown for each of the four protomers in different shades of blue. Locations of very well defined water molecules are shown in red. (*b*) The same color-coding as in (*a*) but rotated by 90°. (*c*) Grouped *B* factor mapped onto the density. The color gradient is from blue to red corresponding to increasing *B* factors. In contrast to *So*BDH2, the *B*-factor distribution is uniform. (*d*) The *Sr*BDH1 structure in cartoon representation. The same view and color-coding are used as in (*a*). (*e*) Enlargement of the C-terminal end of αF with an alternate side-chain conformation of Ser179 and well defined water molecules, shown as red spheres. (*f*) C-terminal end of αH with Phe260 with a characteristic hole in the density for the aromatic ring system. (*g*) Double conformation of Arg74. The water molecule is at a distance of 2.5 Å from the guanidinium function of Arg74 in side-chain conformation Arg74(*B*).

Cryo-grid preparation for *Sr*BDH1 was performed as for *So*BDH2. To ensure that the resolution would not be limited by the sampling of the detector, we decided to increase the magnification during data acquisition. By picking 1 635 690 particle images from 1666 micrographs, we generated a data set of similar size to that for *So*BDH2. Following the same data-processing routine as used for *So*BDH2 yielded a final *Sr*BDH1 reconstruction at 1.88 Å resolution (Table 1 ▸, Supplementary Figs. S4 and S5). This, to the best of our knowledge, is the highest reported resolution of a sub-200 kDa protein solved by single-particle cryoEM. Remarkably, the resolution of the cryoEM structure of apo *Sr*BDH1 is much higher compared with the best resolved crystal structure of *Sr*BDH1–NAD^+^ (PDB entry 6zyz; Chánique *et al.*, 2021 ▸), with four bound NAD^+^ molecules, at 2.27 Å resolution. As assumed, *Sr*BDH1 is arranged as a tetramer (Fig. 4 ▸ and Supplementary Fig. S4). During atomic modeling we followed the same refinement procedure as described for *So*BDH2 with the exception that we used the crystal structure of apo *Sr*BDH1 (PDB entry 6zz0; Chánique *et al.*, 2021 ▸) as the starting model. The crystal structure and cryoEM structure are practically identical (Supplementary Table S3). The density is of outstanding quality, allowing the unambiguous assignment of amino-acid side chains in alternate conformations (Fig. 4 ▸ and Supplementary Fig. S6) and the placement of water molecules.

Almost the entire protein chain could be traced in the cryoEM map, which is reflected by an exceptional atom inclusion level at the moderate contour level of 0.3 for 97% of all backbone atoms and 93% of all non-H atoms. In addition to the first eight residues and the very C-terminal residue (Fig. 2 ▸ *e*), the region from Leu193 to Leu205 is not defined in the density due to the missing NAD^+^ cofactor, as in *So*BDH2 (Fig. 2 ▸ *e* and Supplementary Fig. S7*a* ). In comparison to the available crystal structures of *Sr*BDH1, the total number of built residues is practically identical.

The *Sr*BDH1 model derived from the cryoEM map is virtually identical to the apo-state crystal structure (r.m.s.d. of 0.6 Å for 982 pairs of C^α^ atoms; Supplementary Fig. S7*b* ). At 1.88 Å resolution we could identify 399 water molecules, which uniformly cover the protein surface or are bound in cavities within the protein core. The ratio of water molecules to residues (0.4) is much lower compared with structures determined by X-ray crystallography, where one water molecule per residue is expected at a resolution of 2.0 Å (Carugo & Bordo, 1999 ▸). This discrepancy is explained by the absence of solvent channels in cryoEM structures and the missing local proximity of protein molecules. We observed 34 side chains with a double conformation, corresponding to about 3.5% of all residues. The observed ratio is perfectly in line with a detailed study reporting that 3% of residues present alternate side-chain conformations in protein crystal structures with a resolution between 1.0 and 2.0 Å (Miao & Cao, 2016 ▸).

Since the number of high-resolution cryoEM structures is limited, we wondered whether the Ramachandran *Z*-scores (Hooft *et al.*, 1997 ▸) of our structures (Table 1 ▸) would follow the distribution of Ramachandran *Z* ranges as observed for crystal structures in a similar resolution regime (Sobolev *et al.*, 2020 ▸). The Ramachandran *Z*-scores of the *Sr*BDH1 and *So*BDH2 structures are in the expected region for crystal structures of similar resolution. Notably, we refined the models without Ramachandran restraints, demonstrating that the Ramachandran *Z*-score can also be a valuable measure for cryoEM densities.

## 4. Summary

Structures of homomultimeric plant enzymes are underrepresented in the fast-growing collection of protein structures analyzed by cryoEM. Here, we elucidated the cryoEM structures of two comparatively small plant BDHs to high resolution. Given the molecular mass of the tetrameric complex, here we report the highest resolution achieved by cryoEM so far (Supplementary Table S1), pushing the boundaries of this rapidly developing method.

The new *So*BDH2 structure we describe revealed details of the active-site architecture of the enzyme and allowed comparison to *Sr*BDH1. To our surprise, we could not observe NAD^+^ in the cryoEM structure of *Sr*BDH1, although the protein samples used for crystallization and cryoEM were identical. A possible explanation for this difference could be that in the crystal the cofactor-binding loop is stabilized by crystal contacts and thus may have trapped NAD^+^. Alternatively, vitrification of the sample for cryoEM may have led to the loss of the cofactor.

We attempted to find an explanation why *Sr*BDH1, but not *So*BDH2, could be crystallized. Firstly, *Sr*BDH1 has a considerably higher *T*_m_ compared with *So*BDH2, suggesting a higher fold stability that may be more amenable to crystallization. Furthermore, although the cryoEM structures superimpose with an r.m.s.d. of 1.3 Å for 952 pairs of C^α^ atoms (Supplementary Fig. S7*a* ), local structural differences might have hindered the crystallization of *So*BDH2. The αC helix of *So*BDH2 (Gln52–Gly65) is weakly defined in the density and hence is much more flexible compared with that in *Sr*BDH1 (Figs. 2 ▸ *c*, 2 ▸ *e* and 3 ▸ *c*). Furthermore, in the *Sr*BDH1 structure the αFG helix, upstream of the unresolved loop region, is stabilized by the C-terminal αH helix via hydrophobic contacts. In contrast, the C-terminus is shorter and is not folded in an α-helix in *So*BDH2 (Figs. 2 ▸ *c*, 2 ▸ *e* and Supplementary Fig. S7*a* ). Lastly, we cannot rule out that the NAD^+^ cofactor might stabilize *Sr*BDH1 to a larger extent, and its presence might support the crystallization process, which is not the case for *So*BDH2.

Given the small size of our protein samples and the high particle density on the grids, sufficient data for high-resolution structure analysis could rapidly be acquired, reducing the use of valuable instrument time. Given the high resolution of our structures, model building was greatly facilitated by automated routines, in particular *ARP*/*wARP* *ARPEM* (Chojnowski *et al.*, 2019 ▸) in combination with iterative refinement cycles in *REFMAC*5 (Murshudov *et al.*, 2011 ▸). Moreover, due to the small protein size, real-space refinement and validation was fast.

During the past two decades, X-ray crystallography has been the main structural biochemical method to support drug development. Our observation that high-resolution (≤2.0 Å) structures of rather small proteins can be elucidated by cryoEM in a short time emphasizes the important role that cryoEM has to play in future drug-development efforts, for example using high-throughput applications such as fragment-based screening. Apart from circumventing time-consuming crystallization screening and possible phasing problems, an additional considerable advantage of cryoEM in these and other endeavors is a much-reduced sample consumption compared with crystallography. Likewise, our findings show that cryoEM is already an attractive tool for the structural analysis of enzymes used in green industry.

The availability of high-resolution structural data on newly discovered enzymes is crucial for understanding the molecular basis of their catalytic properties. Furthermore, with this knowledge, characteristics such as stability and selectivity can be improved by rational protein engineering instead of the time-consuming random mutagenesis approaches (Jemli *et al.*, 2016 ▸). Rational design will greatly facilitate the generation of tailor-made enzymes in relatively short time periods. CryoEM is a valuable tool to achieve these goals, as it allows the fast and high-resolution structure determination of enzymes that prove difficult to crystallize.

## 5. Related literature

The following references are cited in the supporting information for this article: Cunha *et al.* (2021 ▸), Fan *et al.* (2019 ▸), Greber *et al.* (2021 ▸), Guntupalli *et al.* (2021 ▸), Herzik *et al.* (2017 ▸, 2019 ▸), Kern *et al.* (2021 ▸), Krissinel & Henrick (2004 ▸), Merk *et al.* (2016 ▸, 2020 ▸), Munir *et al.* (2021 ▸), Nakane *et al.* (2020 ▸) and Zhang *et al.* (2019 ▸).

## Supplementary Material

PDB reference: *Salvia officinialis* borneol dehydrogenase 2, 7o6p

PDB reference: *Salvia rosmarinus* borneol dehydrogenase 1, 7o6q

EMDB reference: *Salvia officinialis* borneol dehydrogenase 2, EMD-12739

EMDB reference: *Salvia rosmarinus* borneol dehydrogenase 1, EMD-12740

Supplementary Figures and Tables. DOI: 10.1107/S205979832101216X/ud5029sup1.pdf

## Figures and Tables

**Table 1:** CryoEM data-collection, refinement and validation statistics

	*Sr*BDH1 (PDB entry 7o6q, EMDB entry EMD-12740)	*So*BDH2 (PDB entry 7o6p, EMDB entry EMD-12739)
Data collection and processing		
Microscope	FEI Titan Krios G3i	FEI Titan Krios G3i
Voltage (keV)	300	300
Camera	Falcon 3EC	Falcon 3EC
Magnification (nominal)	120000	96000
Pixel size at detector (Å per pixel)	0.657	0.832
Total electron exposure (e^−^ Å^−2^)	40	40
Exposure rate (e^−^ per pixel per second)	0.6	0.7
No. of frames collected during exposure	33	33
Defocus range (µm)	0.60–1.6	0.60–1.6
Automation software	*EPU* 2.8.1	*EPU* 2.8.1
No. of micrographs collected	1666	1439
No. of micrographs used	1587	1439
Total No. of extracted particles	1635690	1551724
No. of refined particles	410574	1061307
Final No. of particles	210505	173781
Point-group or helical symmetry parameters	*D*2	*D*2
Resolution (global) (Å)		
FSC 0.143 (unmasked/masked)	2.1/1.88	2.6/2.04
Resolution range (local) (Å)	1.66–30.00	1.84–9.96
Map-sharpening *B* factor (Å^2^)	−46	−43
Map-sharpening methods	Local *B* factor	Local *B* factor
Refinement package	*phenix.real_space_refine*	*phenix.real_space_refine*
Model composition		
Non-H atoms	7999	8276
Protein residues	977	1022
Water molecules	399	268
Model refinement		
Model–map scores		
CC (mask)	0.84	0.86
CC (volume)	0.85	0.83
Average FSC (unmasked/masked)	1.6/1.6	1.7/1.7
Average grouped *B* factors (Å^2^)		
Overall	18.4	34.9
Protein residues	18.3	36.2
Water	19.5	33.3
R.m.s.d. from ideal values		
Bond lengths (Å)	0.010	0.008
Bond angles (°)	0.708	0.582
Validation		
*MolProbity* score	1.5	1.7
*CaBLAM* outliers (%)	0.4	1.5
Clashscore	4.8	6.0
Poor rotamers (%)	1.3	0.9
C^β^ deviations	0	0
*EMRinger* score	7.3	5.8
Ramachandran plot		
Favored (%)	96.8	98.2
Allowed (%)	3.2	1.8
Outliers (%)	0.0	0.0
Ramachandran plot *Z*-score (r.m.s.d.)		
Overall	−1.61 (0.23)	−0.54 (0.23)
Helix	−1.25 (0.19)	−0.01 (0.22)
Sheet	−0.75 (0.36)	0.38 (0.39)
Loop	−0.58 (0.30)	−0.96 (0.26)
