# Supplementary material for: CryoEM analysis of small plant biocatalysts at sub-2 Å resolution
Source: Acta Crystallogr D Struct Biol. 2022 Jan 1;78(Pt 1):113–23. doi: 10.1107/S205979832101216X (PMC8725159; doi:10.1107/S205979832101216X)
Supplement: Supplementary file 1 [file d-78-00113-sup1.pdf]

STRUCTURAL BIOLOGY

Volume 78 (2022)

Supporting information for article:

CryoEM analysis of small plant biocatalysts at sub-2 Å resolution

Nicole Dimos, Carl P. O. Helmer, Andrea M. Chánique, Markus C. Wahl, Robert Kourist, Tarek Hilal and Bernhard Loll

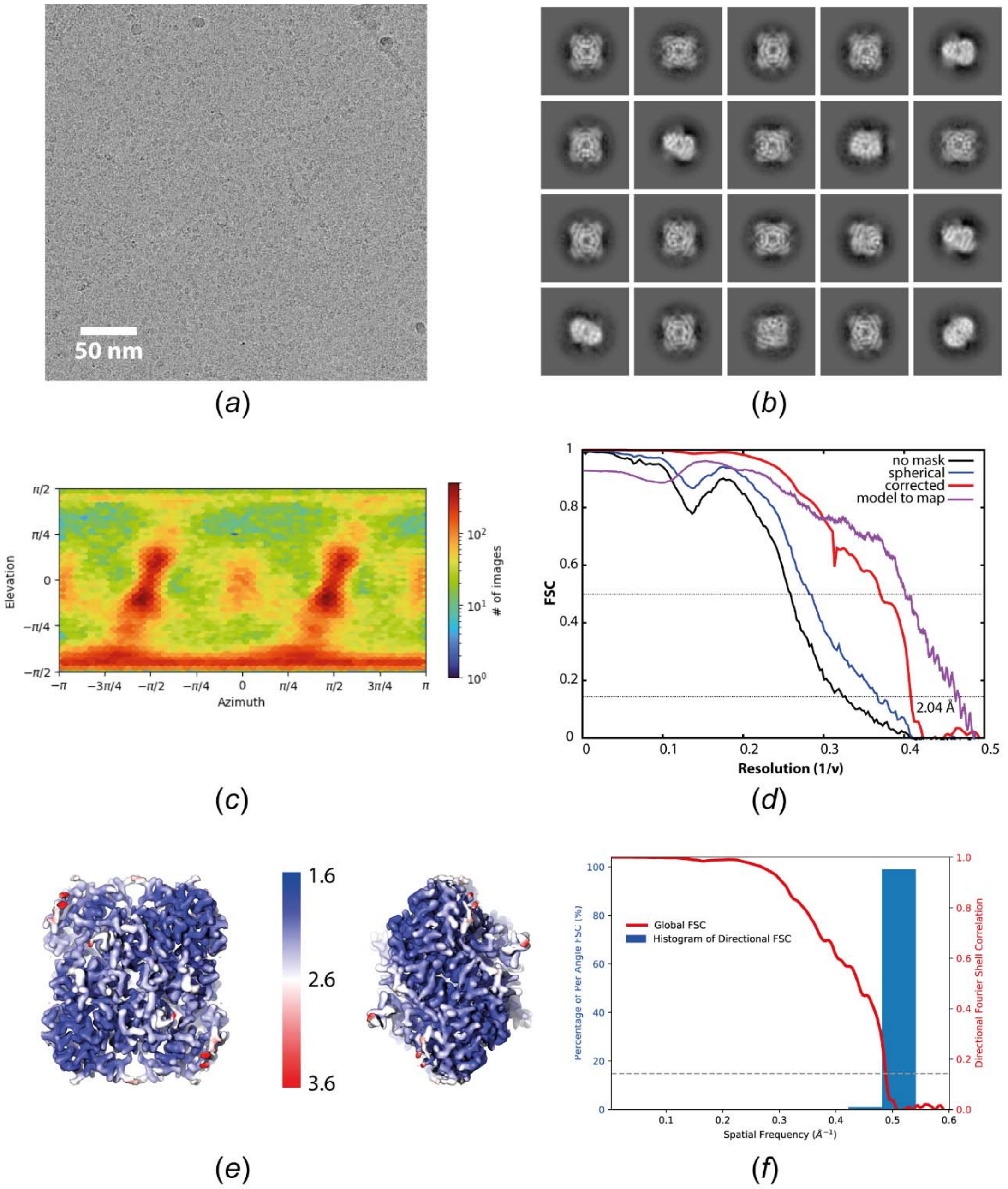

Figure S1 CryoEM analysis of *So*BDH2. (*a*) representative cryoEM micrograph, the scale bar indicates 50 nm spacing. (*b*) selected 2D class averages after reference-free 2D classification with cryoSPARC. Top and side views can be identified, excluding preferential orientation issues. A circular mask of 120 Å diameter was used during classification. (*c*) Viewing direction distribution as determined during non-uniform refinement with cryoSPARC. (*d*) Resolution estimates by fourier-shell correlation using either no mask (black line), a generous spherical mask (blue line) and after solvent correction by phase

randomization (red line). Dashed lines represent FSC(0.5) and FSC(0.143) crossings. Model to map correlation as determined with PHENIX is colored purple. (*e*) Illustration of the local resolution estimation calculated with cryoSPARC for two different views of *So*BDH2 after rotation by 90°. Coloring of the cryoEM density reflects the local resolution ranging from 1.6 Å to 3.6 Å. A major fraction of the structure is resolved well beyond 2 Å, less resolved regions are mainly situated in the periphery of *So*BDH2. (f) results from 3DFSC calculations show an overall good agreement of the directional FSC with the global FSC.

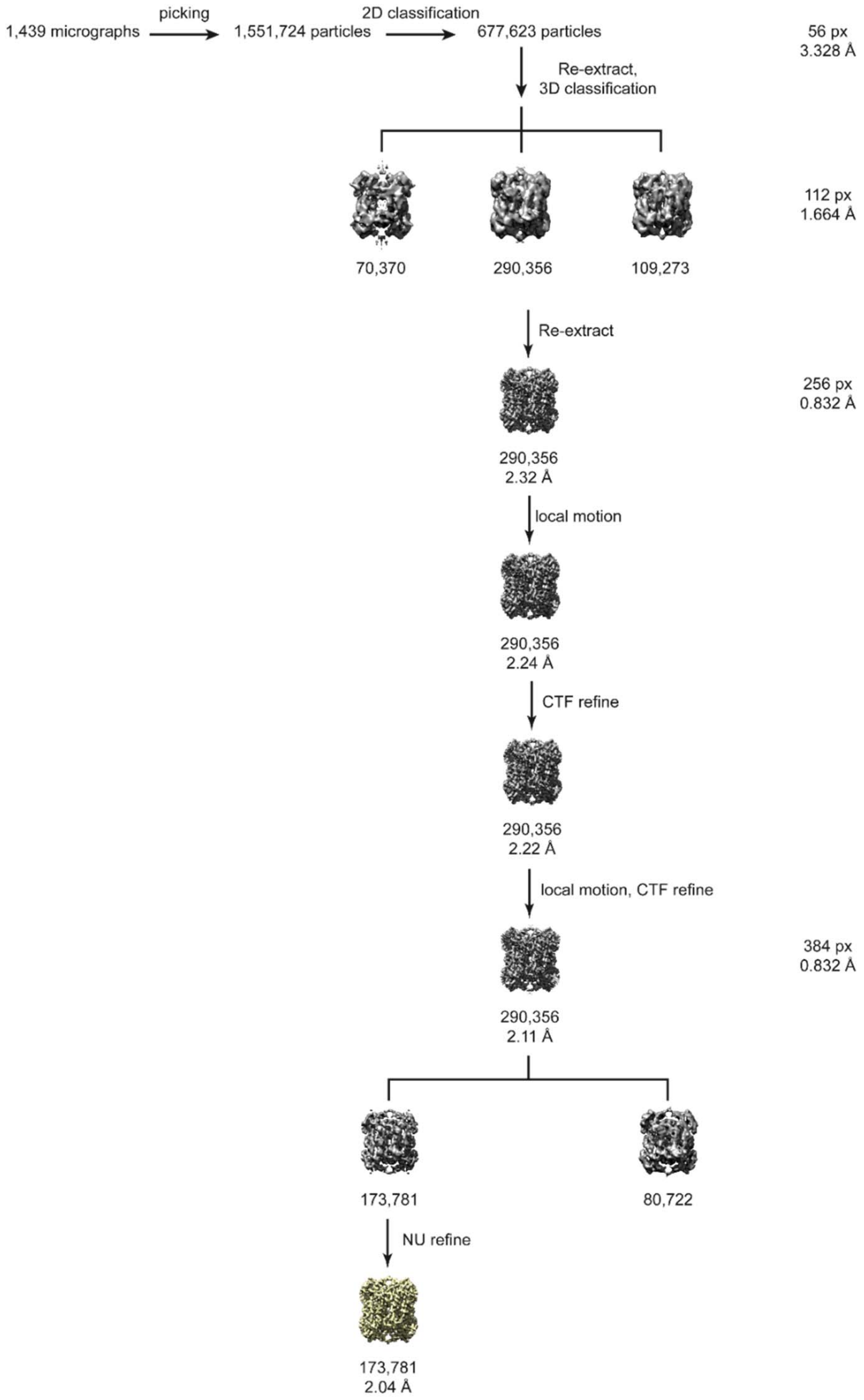

Figure S2 Data processing workflow for the *So*BDH2 dataset. From initially selected 1,439 micrographs ~1.5 M particles were picked and subjected to reference-free 2D classification. ~678k particle images were re-extracted with a box-size of 224 px Fourier-cropped to 112 px giving a pixel-size of 1.664 Å. After 3D classification, a subset of 290,356 particle images was again re-extracted at full resolution with a larger box size of 256 px and homogeneously refined to 2.32 Å resolution. Particle

based local motion correction improved the resolution to 2.24 Å, which could be only marginally improved to 2.22 Å by CTF refinement. Another cycle of local motion correction was applied using a larger extraction box of 384 px to preserve high frequency information of the CTF. Following CTF refinement the resolution improved to 2.11 Å. By heterogeneous refinement, a final subset of 173,781 particle images was selected for homogeneous NU refinement, yielding the final reconstruction at 2.04 Å resolution.

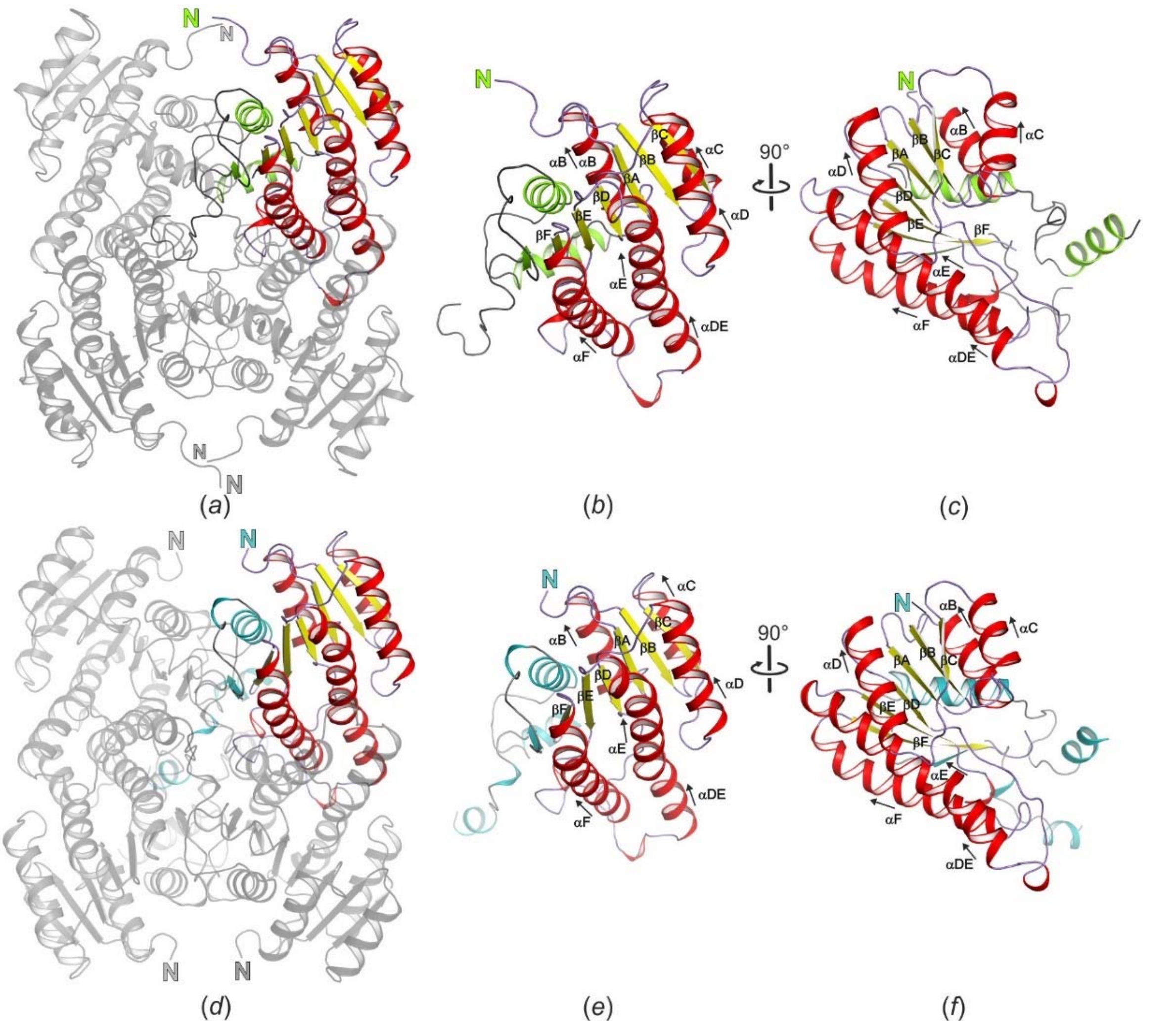

Figure S3 The Rossmann fold in BDHs. (*a*) Same view as in Figure 1a. Tetrameric architecture of *So*BDH2 with three protomers in grey. In one monomer the Rossmann fold is highlighted with yellow β-strands, red α-helices, and purple loop regions. The remaining structure is colored in green and loop regions in gray. (*b*) Identical view as in panel (a), zoom on one protomer. (*c*) view of (b) rotated by 90 °. (*d*) Same view as in Figure 3a. Tetrameric architecture of *Sr*BDH1 with three protomers in grey. In one monomer the Rossmann fold is highlighted with yellow β-strands, red α-helices, and purple loop regions. The remaining structure is colored in teal and loop regions in gray. (*e*) Identical view as in panel (a), zoom on one protomer. (*f*) view of (e) rotated by 90 °.

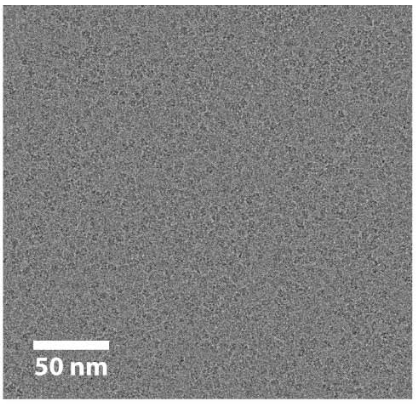

(*a*)

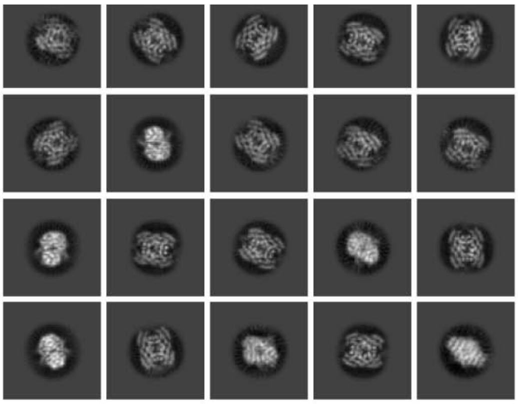

(*b*)

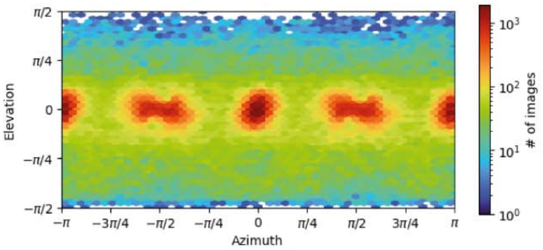

(*c*)

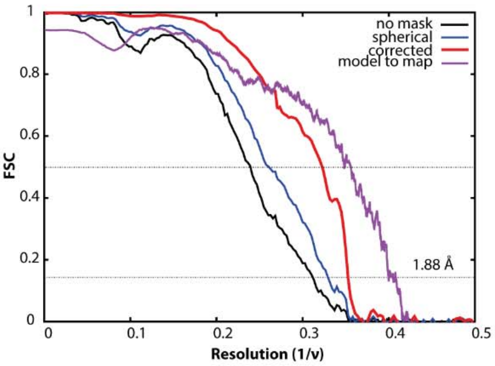

(*d*)

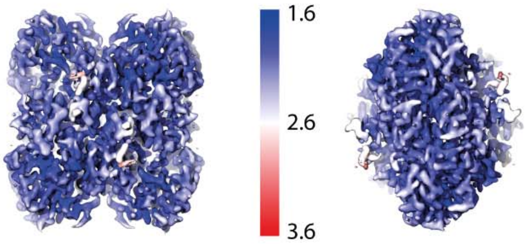

(*e*)

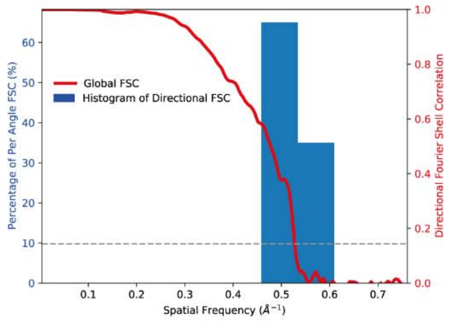

(*f*)

Figure S4 CryoEM analysis of *Sr*BDH1. (*a*) Representative cryoEM micrograph, the scale bar indicates 50 nm spacing. (*b*) selected 2D class averages after reference-free 2D classification with cryoSPARC. As for *So*BDH2, top and side views can be identified. A circular mask of 100 Å diameter was used during classification. (***c***) Viewing direction distribution as determined during non-uniform refinement with cryoSPARC. (*d*) Resolution estimates by Fourier-shell correlation using either no mask (black line), a generous spherical mask (blue line) and after solvent correction by phase randomization (red line).

Dashed lines represent FSC(0.5) and FSC(0.143) crossings. Model to map correlation as determined with PHENIX is colored purple. (*e*) Illustration of the local resolution estimation calculated with cryoSPARC for two different views of *Sr*BDH1 after rotation by 90°. Coloring of the cryoEM density reflects the local resolution ranging from 1.6 to 3.6 Å. The vast majority of the structure is resolved well beyond 2 Å. (*f*) 3DFSC calculations confirm that the global FSC falls in between the only two existing bins of directional FSCs. The directional resolution anisotropy did not result in obvious peculiarities of the reconstruction.

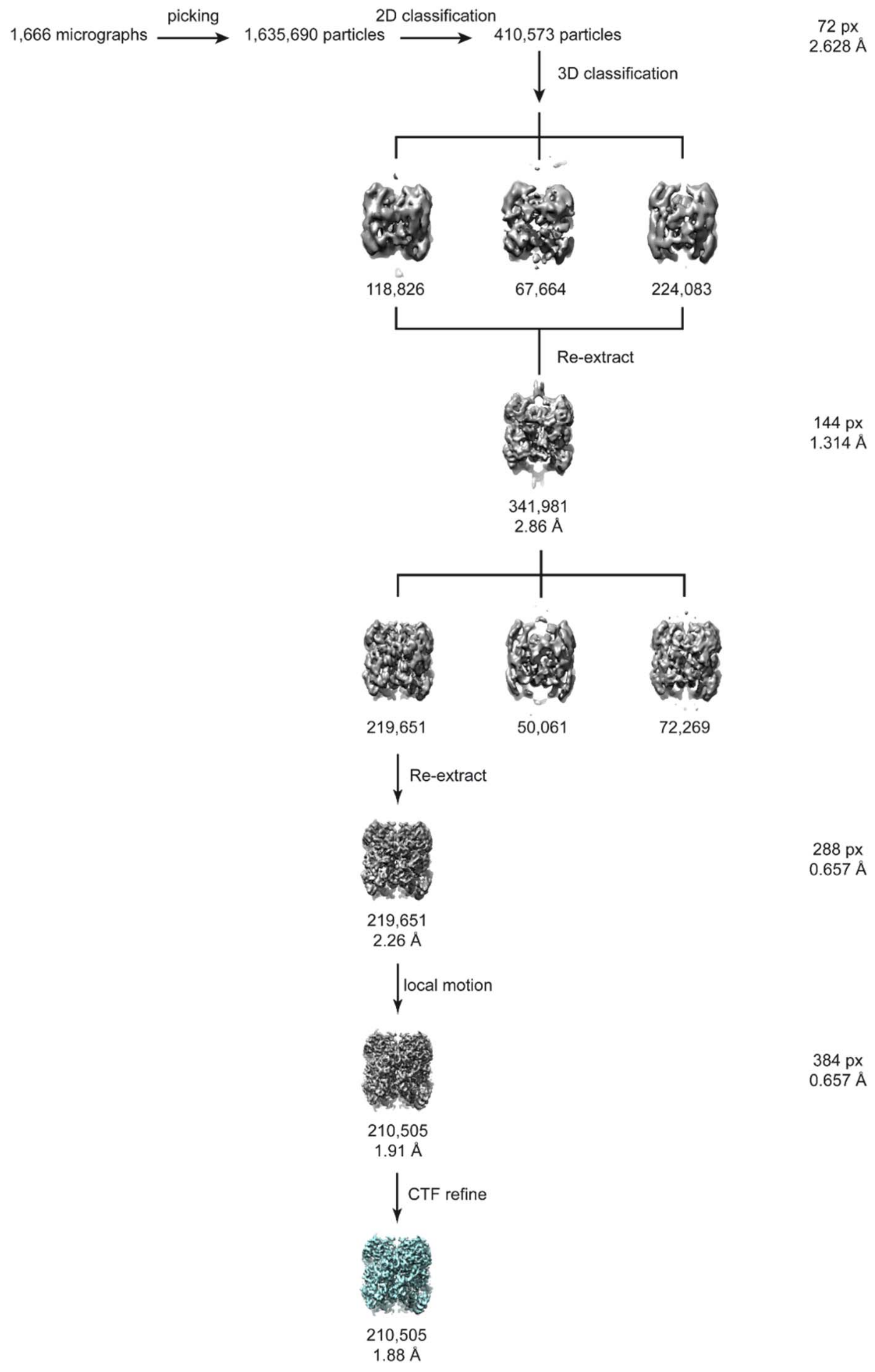

Figure S5 Data analysis of the *Sr*BDH1 dataset. Using the *So*BDH2 structure as reference, ~1.6 M particles were automatically picked from 1,666 micrographs with cryoSPARC. Iterations of 2D classification were applied to select 410,573 particle images for heterogeneous 3D classification. A subset of 341,981 was re-extracted with a box-size of 288 px, fourier-cropped to 144 px and homogeneously refined to 2.86 Å resolution. After another heterogeneous refinement using 3 classes, 219,651 particles were re-extracted at full resolution (0.657 Å/pix) yielding a reconstruction of 2.16 Å. Local motion

correction was applied, after which 210,505 particles were re-extracted with a box size of 384 px and homogeneously refined to 1.91 Å resolution. CTF refinement followed by NU refinement generated the final reconstruction with 1.88 Å resolution (cyan).

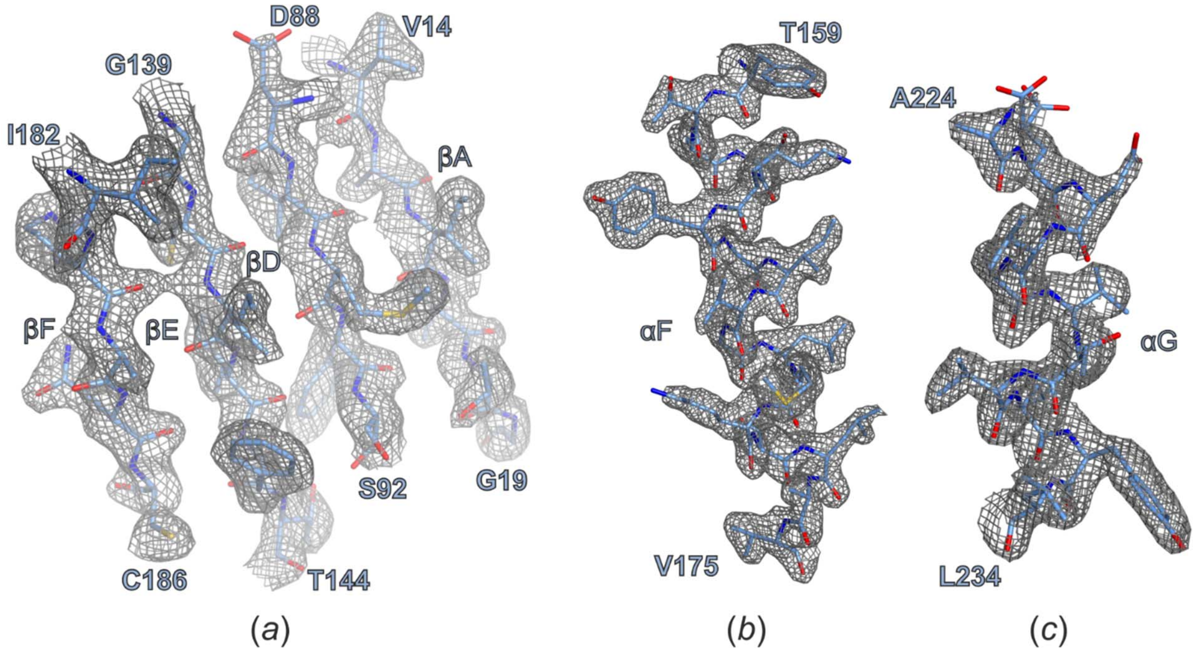

Figure S6 Examples of the high-quality electron volumes of *Sr*BDH1. (*a*) Zoom on the central β-sheet. (*b*) and (*c*) examples of two α-helices.

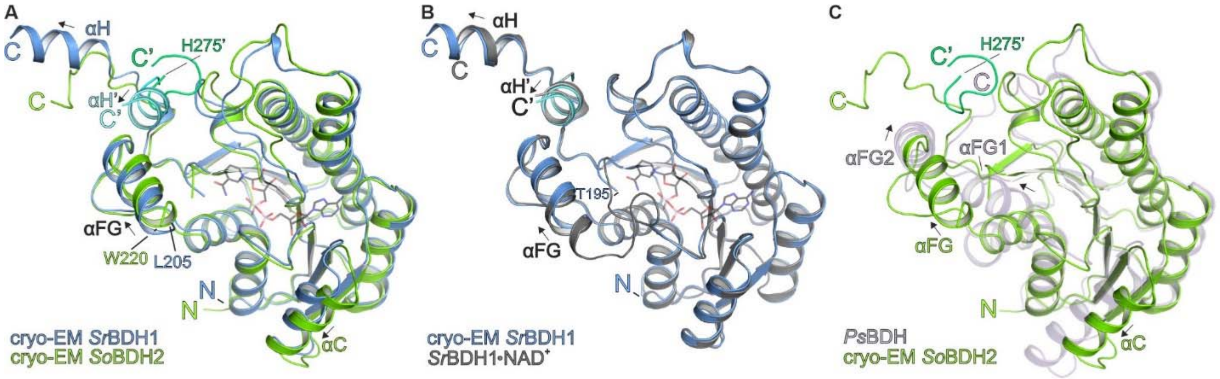

Figure S7 Structural comparison of related BDH structures focusing on the substrate/cofactor binding site. Only one protomer of the tetrameric complexes is shown. The proteins are shown in cartoon representation. (*a*) Superposition of the cryoEM structures of *Sr*BDH1, drawn in blue, as well as *So*BDH2, drawn in green cartoon. The C-terminal αH helix of another protomer completes the substrate binding site. The NAD^+ molecule is drawn in black, obtained by a superposition with the crystal structure of *Sr*BDH1•NAD^+ / PO/OH (PDB ID 6ZYZ) (Chánique *et al.*, 2021). Structural differences can be seen in particular for the C-terminus and the αC helix. (*b*) Superposition of the cryoEM structure of *Sr*BDH1 drawn in blue and the crystal structure of *Sr*BDH1•NAD^+ / PO/OH (PDB ID 6ZYZ (Chánique *et al.*, 2021)) drawn in gray. Binding of NAD^+ leads to stabilization of the loop region upstream of helix αFG and the helix itself. (*c*) Superposition of the cryoEM structure of *So*BDH2 and the crystal structure of *Ps*BDH PDB ID 6M5N (Khine *et al.*, 2020)) shown in light purple. Major structural differences are observed for the C-terminal portion of the protein.

Table S1 Overview of selected cryoEM structures with the highest achieved resolution.

The summary does not include larger multi-subunit complexes. Membrane proteins incorporated in nanodiscs are indicated with an asterisk.

enzyme	organism	assembly	resolution [Å]	total M_r [kDa]	EMDB ID	reference
apoferritin	*H. sapiens*	24-mer	1.15	480	11668	(Yip *et al.*, 2020)
β_3 GABAA receptor*	*H. sapiens*	pentamer	1.7	200	11657	(Nakane *et al.*, 2020)
β-galactosidase	*E. coli*	tetramer	1.8	465	21995	(Merk *et al.*, 2020)
BDH1	*S. rosmarinus*	tetramer	1.88	120	12740	This study
urease	*H. pylori*	dodecamer	2.04	1100	11233	(Cunha *et al.*, 2021)
BDH2	*S. officinalis*	tetramer	2.04	129	12739	This study
ORF3a/apolipo*	SARS-CoV-2	tetramer	2.08	114	22898	(Kern *et al.*, 2021)
CDK-activating kinase	*H. sapiens*	dimer	2.51	119	12042	(Greber *et al.*, 2021)
aldolase	*O. cuniculus*	tetramer	2.6	150	8743	(Herzik *et al.*, 2017)
catalase-peroxidase	*M. tuberculosis*	dimer	2.68	161	11776	(Munir *et al.*, 2021)
alcohol dehydrogenase	*S. carlsbergensis*	tetramer	2.7	147	22807	(Guntupalli *et al.*, 2021)
methemoglobin	*H. sapiens*	tetramer	2.8	64	0407	(Herzik *et al.*, 2019)
lactate dehydrogenase	*G. gallus*	tetramer	2.8	144	8191	(Merk *et al.*, 2016)
alcohol dehydrogenase	*E. caballus*	dimer	2.9	82	0406	(Herzik *et al.*, 2019)
biotin-bound streptavidin	*S. avidinii*	tetramer	3.2	52	0689	(Fan *et al.*, 2019)
cytotoxin A	*H. pylori*	hexameric	3.2	530	0542	(Zhang *et al.*, 2019)

isocitrate dehydrogenase	*H. sapiens*	dimer	3.8	93	8193	(Merk *et al.*, 2016)
catalytic subunit protein kinase A	*M. musculus*	monomer	6.0	43	0409	(Herzik *et al.*, 2019)

Table S2 Comparison of the performance of different automated model building programs.

ARP/wARP – ARPEM(Chojnowski *et al.*, 2019), phenix.map_to_model(Terwilliger *et al.*, 2018), as well as Buccaneer(Hoh *et al.*, 2020). Green numbers refer to the structure of *So*BDH2 and blue numbers to *Sr*BDH1

	**ARP/wARP ARPEM**	**Phenix map_to_model**	**CCPEM Buccaneer**	**Final model**
Total number of residues		**1212** / **1160**		
Residues built	**1001** / **963**	**880** / **828**	**1159** / **972**	**1022** / **977**
Residues sequenced	**922** / **921**	**880** /	**1047** / **955**	**1022** / **977**
Completeness by residues built [%]	**82.0** / **83.0**	**72.6** /	**91.5** / **83.8**	**84.5** / **84.2**

Table S3 Structural comparison of the three different crystal structures of *Sr*BDH1.

*Sr*BDH1 apo (PDB ID 6ZZ0), *Sr*BDH1•NAD$^+$ / high salt (PDB ID 6ZZT) with one bound NAD$^+$ as well as *Sr*BDH1•NAD$^+$ / PO/OH (PDB ID 6ZYZ) with four bound NAD$^+$ molecules (Chánique *et al.*, 2021) as well as the two cryoEM structures of *Sr*BDH1 and *So*BDH2. R.m.s.d. for pairs of Cα-atoms calculated with SSM (Krissinel & Henrick, 2004) as implemented in COOT (Casañal *et al.*, 2020).

	***Sr*BDH1 apo**	***Sr*BDH1•NAD$^+$ / high salt**	***Sr*BDH1•NAD$^+$ / PO/OH**	**cryoEM *Sr*BDH1**	**cryoEM *So*BDH2**
***Sr*BDH1 apo**					
***Sr*BDH1•NAD$^+$ / high salt**	0.48				
***Sr*BDH1•NAD$^+$ / PO/OH**	0.56	0.52			
**cryoEM *Sr*BDH1**	0.57	0.49	0.39		
**cryoEM *So*BDH2**	1.44	1.39	1.39	1.15	

References

Casañal, A., Lohkamp, B. & Emsley, P. (2020). *Protein Sci.* **29**, 1069-1078.

Chánique, A., Dimos, N., Drienovska, I., Calderinia, E., Pantína, M. P., Helmer, C. P. O., Hilal, T., Hofer, M., Sieber, V., Parraf, L. P., Loll, B. & Kourist, R. (2021). *ChemCatChem* **13**, 2262-2277.

Chojnowski, G., Pereira, J. & Lamzin, V. S. (2019). *Acta Crystallogr D Struct Biol* **75**, 753-763.

Cunha, E. S., Chen, X., Sanz-Gaitero, M., Mills, D. J. & Luecke, H. (2021). *Nat Commun* **12**, 230.

Fan, X., Wang, J., Zhang, X., Yang, Z., Zhang, J. C., Zhao, L., Peng, H. L., Lei, J. & Wang, H. W. (2019). *Nat Commun* **10**, 2386.

Greber, B. J., Remis, J., Ali, S. & Nogales, E. (2021). *Biophys J.*

Guntupalli, S. R., Li, Z., Chang, L., Plapp, B. V. & Subramanian, R. (2021). *Biochemistry*.

Herzik, M. A., Jr., Wu, M. & Lander, G. C. (2017). *Nat Methods* **14**, 1075-1078.

Herzik, M. A., Jr., Wu, M. & Lander, G. C. (2019). *Nat Commun* **10**, 1032.

Hoh, S. W., Burnley, T. & Cowtan, K. (2020). *Acta Crystallogr D Struct Biol* **76**, 531-541.

Kern, D. M., Sorum, B., Hoel, C. M., Sridharan, S., Remis, J. P., Toso, D. B. & Brohawn, S. G. (2020). Cryo-EM structure of the SARS-CoV-2 3a ion channel in lipid nanodiscs, bioRxiv.

Khine, A. A., Chen, H. P., Huang, K. F. & Ko, T. P. (2020). *Acta Crystallogr F Struct Biol Commun* **76**, 309-313.

Krissinel, E. & Henrick, K. (2004). *Acta Crystallogr D Biol Crystallogr* **60**, 2256-2268.

Merk, A., Bartesaghi, A., Banerjee, S., Falconieri, V., Rao, P., Davis, M. I., Pragani, R., Boxer, M. B., Earl, L. A., Milne, J. L. S. & Subramaniam, S. (2016). *Cell* **165**, 1698-1707.

Merk, A., Fukumura, T., Zhu, X., Darling, J. E., Grisshammer, R., Ognjenovic, J. & Subramaniam, S. (2020). *IUCrJ* **7**, 639-643.

Munir, A., Wilson, M. T., Hardwick, S. W., Chirgadze, D. Y., Worrall, J. A. R., Blundell, T. L. & Chaplin, A. K. (2021). *Structure*.

Nakane, T., Kotecha, A., Sente, A., McMullan, G., Masiulis, S., Brown, P., Grigoras, I. T., Malinauskaite, L., Malinauskas, T., Miehling, J., Uchanski, T., Yu, L., Karia, D., Pechnikova, E. V., de Jong, E., Keizer, J., Bischoff, M., McCormack, J., Tiemeijer, P., Hardwick, S. W., Chirgadze, D. Y., Murshudov, G., Aricescu, A. R. & Scheres, S. H. W. (2020). *Nature* **587**, 152-156.

Terwilliger, T. C., Adams, P. D., Afonine, P. V. & Sobolev, O. V. (2018). *Nat Methods* **15**, 905-908.

Yip, K. M., Fischer, N., Paknia, E., Chari, A. & Stark, H. (2020). *Nature* **587**, 157-161.

Zhang, K. M., Zhang, H. W., Li, S. S., Pintilie, G. D., Mou, T. C., Gao, Y. Z., Zhang, Q. F., van den Bedeme, H., Schmid, M. F., Au, S. W. N. & Chiu, W. (2019). *P Natl Acad Sci USA* **116**, 6800-6805.
